# Multiple anthropometric and nutritional deficiencies in young children in Ethiopia: a multi-level analysis based on a nationally representative data

**DOI:** 10.1186/s12887-020-02467-1

**Published:** 2021-01-05

**Authors:** Nigatu Regassa Geda, Cindy Xin Feng, Carol J. Henry, Rein Lepnurm, Bonnie Janzen, Susan J. Whiting

**Affiliations:** 1 Center for Population Studies, College of Development Studies, Addis Ababa University, Sidist Kilo Campus, PO Box 1176, Addis Ababa, Ethiopia; 2 School of Public Health, Health Science E-wing, University of Saskatchewan, 104 Clinic Place, S7N 2Z4 Saskatoon, SK Canada; 3 Department of Community Health and Epidemiology, Faculty of Medicine, Dalhousie University, Halifax, Canada; 4 College of Pharmacy and Nutrition, University of Saskatchewan, Health Sciences A-wing, 107 Wiggins Road, S7N 5E5 Saskatoon, SK Canada; 5 Dept of Community Health & Epidemiology, Collège of Medicine, University of Saskatchewan, Saskatoon, Canada

**Keywords:** Anemia, Nutritional deficiencies, Undernutrition, Stunting, Wasting, Underweight

## Abstract

**Background:**

In Ethiopia, child undernutrition and anemia are major public health concerns, resulting in increased childhood morbidity and mortality. Despite progress made to reduce the prevalence of malnutrition (especially stunting) from 50% in 2000 to 38% in 2016, little is known about the magnitude and risk factors for concurrent nutritional deficiencies in Ethiopia.

**Methods:**

Analysis for this study was based on a total sample of 9218 children aged 6–59 months drawn from the Ethiopian Demographic and Health Survey (EDHS) conducted in the year 2016. The study used two outcome variables: Multiple nutrition deficit index formed by combining stunting, underweight, wasting and anemia status; and a concurrent stunting and anemia (CAS) index. Two mixed effect regression models, Poisson and Logistic, were used to identify the key risk factors of the two outcome variables, respectively.

**Results:**

The proportion of children with stunting (length-for-age), underweight (weight-for-age) and wasting children (weight-for-length) was 38%, 25.2% and 9.4%, respectively. About 58% of the children had anemia. The prevalence of children with concurrent stunting and anemia children was 24.8%. Our results showed that the risks of multiple nutritional problems were determined by a range of individual, household and behavioral factors including: sex of the child, age of the child, birth order, parity, parental education, religion, household wealth index and type of family structure. The proximate variables (hygiene and sanitation score, feeding practice, and child health service utilization score) were also found to exert a strong influence on the risk of multiple nutritional deficiencies. The likelihood of co-occurrence of stunting and anemia was determined by certain individual and household factors, including sex of the child, age of the child, maternal education, household asset based wealth, religion and household hygiene and sanitation.

**Conclusions:**

This study underscores the importance of improving parental education, household wealth, hygiene and sanitation conditions, promoting feeding practice and child health service utilization. Also, any nutrition sensitive and specific intervention should consider a child’s characteristics such as his/her age, gender and birth order.

## Background

Worldwide undernutrition restricts fetal growth causing stunting and wasting; furthermore, micronutrient deficiencies are responsible for the annual death of 3.1 million children under five years of age (under-5) [1]. It is estimated that 45% of deaths among children under-5 are linked to undernutrition (WHO,2018). Stunting is a chronic form of malnutrition, a result of long-term nutritional deprivation. It is defined as height-for-age < -2 standard deviation (SD) of the World Health Organization’s growth standard median [2]. Wasting represents thinness, i.e., weight-for-height < -2 SD, and underweight reflects low body mass relative to chronological age, i.e., weight-for-age <-2 SD [2]. In Sub-Saharan Africa, the prevalence of underweight, stunting and wasting in children under-5 was 21%, 40% and 9%, respectively [3].

Previous studies around the world reported the heightened risk of mortality for children with multiple nutrition deficits [4–6]. For instance, a child with wasting and stunting is 12 times more likely to die than a child without these anthropometric deficiencies [7]. Clustering of nutrition problems can occur national, regional and individual levels. This indicates that there is considerable overlap of risk factors/determinants [1, 4, 8]. Some previous studies confirmed that anemia and stunting share many common risk factors [4, 9, 10].

In Ethiopia, in 2012, the prevalence of stunting, underweight and wasting among under-5 children were reported as 44.4%, 28.7% and 9.7%, respectively [11]. As of 2016, 38% of children under-5 had stunting. Stunting in children was higher in rural areas (40%) than in urban areas (25%) [12]. About 58% of under-5 children in Ethiopia had anemia [12]. A recent assessment by the International Fund for Agricultural Development/IFAD confirmed that Ethiopia has huge structured inequalities (deep rooted) in undernutrition of children both within households and community levels [13].

The very few studies conducted on this subject in Ethiopia are limited to investigating the determinants of one of the three types of child malnutrition (i.e., stunting, wasting or underweight), and most of which were conducted using micro-level data [5, 14, 15]. Nevertheless, children are likely to be affected by double or even multiple forms of nutritional problems, which have not been investigated well [4, 6]. For instance, a child could be underweight and anemic at the same time, mostly driven by certain risk factors. This undoubtedly poses significant challenges to child survival [4, 5, 16]. A study by Shimeles and colleagues [4] was the only study on a nationally representative data reporting considerable co-occurrence of anemia and stunting (CAS) in Ethiopia [4]. This study, however, addressed only children 6–23 months of age and did not address undernutrition in its entirety.

The present study aims to examine the risk factors of co-occurrence of undernutrition and anemia among children of age 6–59 months in Ethiopia based on nationally representative data.

## Methods

### The study context

The most recent estimate of the World Bank report [17] indicates that Ethiopia has a population of 109 million, making it the second-most populous nation in Africa after Nigeria [17]. According to the report, the country is one of the poorest, with an annual per capita income of $790 [17]. Administratively, Ethiopia is a Federal Democratic Republic with nine autonomous Regional States, each divided into zones, districts and sub-districts/ kebeles [18]. Agriculture has been the main driver for the fast-growing Ethiopian economy, responsible for 85% of total employment [13]. Although the rapid economic growth is attributed to the enhancing productivity of agriculture, particularly of crop production but chronic malnutrition (stunting) of children remains unacceptably high. Considering the new Sustainable Development Goals (SDGs), nutrition has been recognized as a major need for sustainable development [13].

The government of Ethiopia has developed various development plans and strategies to increase food security, improve nutrition and reduce poverty [18–20]. The National Nutrition Program II targeted implementation of both nutrition-sensitive and non-nutrition sensitive interventions to significantly improve maternal and child nutrition in the country.

### Data sources

We used data from the Ethiopian Demographic and Health Surveys (EDHS) for 2016. The 2016 survey is one of a series of nationally representative samples, conducted for the fourth time since 2000. The EDHS are cross-sectional data containing comparable household and individual information about sociodemographic characteristics and health indicators such as maternal and child health and nutrition. The EDHS surveys have been carried out nationally by the Central Statistical Agency (CSA) under the guidance of the Ministry of Health (MOH). The data were extracted from the children’s file containing entries for that under-5. Infants below six months of age were excluded since EDHS did not collect data on hemoglobin level for this age group. A total of 9218 children aged 6–59 months was extracted from the dataset for final analysis. As the data were well imputed by the Central Statistics Authority (CSA) of Ethiopia and ICF (the data owners), the overall missing values were limited to 5.8%. The rows with the missing values were excluded from the entire analysis.

### Ethical clearance

The EDHS surveys are well-established, nationally representative data. They are respected global initiatives conducted with appropriate permission from the Ethiopian government and informed consent from subjects. ICF International (U.S.) and the Central Statistics Authority (Ethiopia) granted permission for the use of EDHS. Ethical approval was also received by the University of Saskatchewan, Behavioral Research Ethics Committee.

### Variables and measures

The Ethiopian Demographic and Health Surveys collected information on the health and nutritional status of children. Categorization of undernutrition of children was done using height-for-age (HAZ), weight-for-age (WAZ) and weight-for-height (WHZ) SDs from WHO, also known as z-scores to determine stunting, under-weight, and wasting, respectively [2, 21]. Anemia status was defined by hemoglobin < 11 g/dL [10], and the measure was adjusted for altitude to account for most Ethiopians living at high altitudes where hemoglobin levels are normally higher than at sea level, making true anemia difficult to detect [10]. The present study used two different outcome variables: the number of each of the four possible nutritional problems and the presence of concurrent stunting and anemia (CAS). In the primary analysis, a coding of 1 was used if a child had any of the three anthropometric deficits (stunting, underweight, wasting) or anemia, and “0” if the child experienced none of the four nutritional problems. For the secondary analysis, CAS was the outcome variable. For the CAS, 1 was coded if a child was both anemic and stunted at the same time, and 0 otherwise.

The selection of the explanatory variables was made based on the review of literature, availability of the variable in the data set, and statistical plausibility. The factors influencing multiple anthropometric deficit and CAS were broadly classified as maternal and child characteristics (maternal education, autonomy, maternal parity, maternal age, child’s age, child’s sex, child’s birth order); household factors (the type of family structure, religion, household wealth ); child care practices (feeding practices, child health service utilization score, hygiene and sanitation practice score); and community-level variables ( mean maternal education and wealth at cluster level, and type of residence).

Scores were constructed for some of the potential predictors by combining different variables. For instance, the hygiene and sanitation score was measured by combining responses of household ownership of facilities that ensure hygienic separation of human excreta from human contact (which include flush or pour-flush toilet/latrine, piped sewer system, septic tank, pit latrine, Ventilated Improved pit (VIP) latrine, pit latrine with slab and composting toilet ) [22], hand washing and access to drinking water. The value for the hygiene and sanitation score ranged between 0 and 6. The child health service utilization score was constructed from six dichotomous responses (Antenatal Care/ANC, delivery care, postnatal care, vitamin A, iron supplementation and deworming pills), each coded as 0 or 1. Adding these values for each respondent yielded a score ranging between 0 and 6. The diet diversity score (DDS) was measured based on the consumption of the seven food groups (0 = no, yes = 1) according to the WHO’s IYCF guidelines [23]. These food groups are: (i) grains, roots, and tubers; (ii) flesh foods (meat, fish, poultry and liver/organ meats); (iii) legumes and nuts; (iv) vitamin A rich fruits and vegetables; (v) eggs vi) dairy products (milk, yogurt, cheese); (vii) other fruits and vegetables [23].The DDS score was obtained by summing up the binary responses, and it ranges from 0 to 7, where a higher score represents the higher level of diet diversification.

Household wealth was used as a proxy to household income and was estimated in the DHS with an asset-based index that combined information about ownership of consumer goods and housing quality. It was sorted into three categories for purposes of analysis: poorer, middle, and richer. Similarly, maternal autonomy was measured based on five responses related to her decision making on important household purchases, childcare and mobility. The remaining explanatory variables (such as sex and age of the child, family structure, breastfeeding, and frequency of access to media) were used as coded in the original data.

### Statistical analysis

We analyzed the data using STATA version 12 [24]. All analyses were weighted for the sampling probabilities and considered the stratification and clustering nature of the data. Descriptive analysis was used to examine the characteristics of the study sample.

The DHS data are clustered, i.e., mothers are nested within households, and households are nested within clusters. As such, mothers within the same cluster may be more like each other than mothers in the rest of the clusters. This violates the assumption of independence of observations across the clusters, and hence, limits the use of conventional regression [25]. For the present analysis, the enumeration areas/EAs were used for clustering women respondents. Mixed-effects Poisson regression was used (for the count outcome variable) and mixed-effect logistic regression model (for CAS) to test the effect sizes of individual, household, and community factors. Multicollinearity between the potential predictors was checked using tolerance test, variance inflation factors. To achieve a parsimonious model, a bivariate analysis was first conducted, and all potential predictors which were statistically associated with the outcomes with a p-value < 0.20 were subsequently included in the multivariable analysis. The Akaike Information Criterion (AIC) was used as model selection criteria. In the final model, a p-value of < 0.05 was used to define statistical significance. The model fit was checked using the ratio of Deviance and Degree of Freedom (DF), i.e., Deviance/ DF [26].

## Results

### Anthropometric and nutritional status

Table 1 presents the distribution of the proportion of children by nutritional status. The computed overall prevalence of undernutrition (stunting, underweight and wasting) and anemia among the study participants (6–59 months) were high: stunting (40.7%), underweight (25.2%), wasting (9.4%) and anemia (57.6%).

**Table 1:** Proportion of under-5 children with stunting, wasting, underweight and anemia by selected background characteristics, Ethiopia

Characteristics^**a**^	Stunting N (%)	Underweight N (%)	Wasting N (%)	Anemia ***N***= (%)
**Sex of the child**				
Male	1931 (43.4)	1209 (27.1)	421 (9.4)	2534 (57.7)
Female	1575 (37.9)	965 (23.2)	388 (9.3)	2345 (57.5)
**Place of residence**				
Urban	262 (28.4)	134 (14.5)	80 (8.7)	424 (49.4)
Rural	3244 (42.2)	2040 (26.5)	729 (9.5)	4455 (58.5)
**Age of mother**				
15-24	813 (37.9)	490 (22.7)	221 (10.3)	1106 (62.9)
25-34	1920 (36.8)	1234 (23.6)	487 (9.3)	2661 (57.9)
34+	947 (39.9)	590 (24.8)	261 (10.9)	1115 (52.3)
**Religion**				
Orthodox	1281 (43.1)	748 (25.1)	232 (7.8)	1384 (47.5)
Muslim	1365 (39.2)	918 (26.4)	387 (11.1)	2314 (67.5)
Others	859 (39.9)	507 (23.5)	190 (8.8)	1180 (55.3)
**Education of mothers**				
No education	2511 (43.8)	1663 (28.9)	592 (10.3)	3331 (58.6)
Primary level	873 (37.8)	447 (19.4)	179 (7.8)	1295 (56.9)
Secondary and above	123 (821.7)	63 (11.2)	38 (6.7)	252 (48.8)
**Education of husband**				
No education	1964 (45.0)	1292 (29.5)	457 (10.4)	2542 (58.7)
Primary	1278 (38.7)	744 (22.5)	279 (8.4)	1876 (57.3)
Secondary and higher	263 (28.1)	137 (14.6)	74 (7.9)	460 (52.8)
**Household size**				
1-3 members	346 (44.0)	204 (26.0)	76 (9.7)	472 (61.5)
4-7 members	1764 (39.7)	1078 (24.2)	372 (8.4)	2412 (55.3)
7+	1396 (41.3)	891 (26.3)	361 (10.6)	1995 (59.7)
**Wealth index**				
Poorest	1879 (46.8)	1268 (31.5)	457 (11.3)	2505 (63.0)
Poorer	742 (40.5)	441 (24.1)	170 (9.3)	980 (53.8)
Middle	886 (32.0)	464 (16.8)	182 (6.6)	1393 (52.0)

^a^All analysis were weighted

It is noted that child stunting and underweight were both higher among male children compared to females; 1931 (43.4%) for stunting and 1209 (27.1%) for underweight in males compared to 1575 (37.9%) and 965 (23.2%) in females. There was no sex difference in wasting or anemia prevalence. The prevalence of child undernutrition was much higher in rural areas, and the difference was more pronounced for stunting and underweight. Compared to Orthodox Christian and other religions, children from Muslims households were more prone to experiencing underweight, wasting and anemia, but not stunting. There is noticeable differences in nutritional problems across maternal education groups; for example, all prevalences are highest among children of mothers with no education. There is a notable consistent decline in the prevalence of stunting, wasting, underweight and anemia as we move from the poorest/poorer to the richest/richer wealth groups.

### Multiple nutritional deficit and CAS

As stated in Methods section, the first outcome variable, multiple nutritional deficit, was measured by combining the four nutritional problems. It is a count variable, ranging from 0 to 4, that indicates the number of nutritional problems a single child experienced. The outcome variable has a positively skewed distribution with large proportion of zeros.

**Fig. 1:**
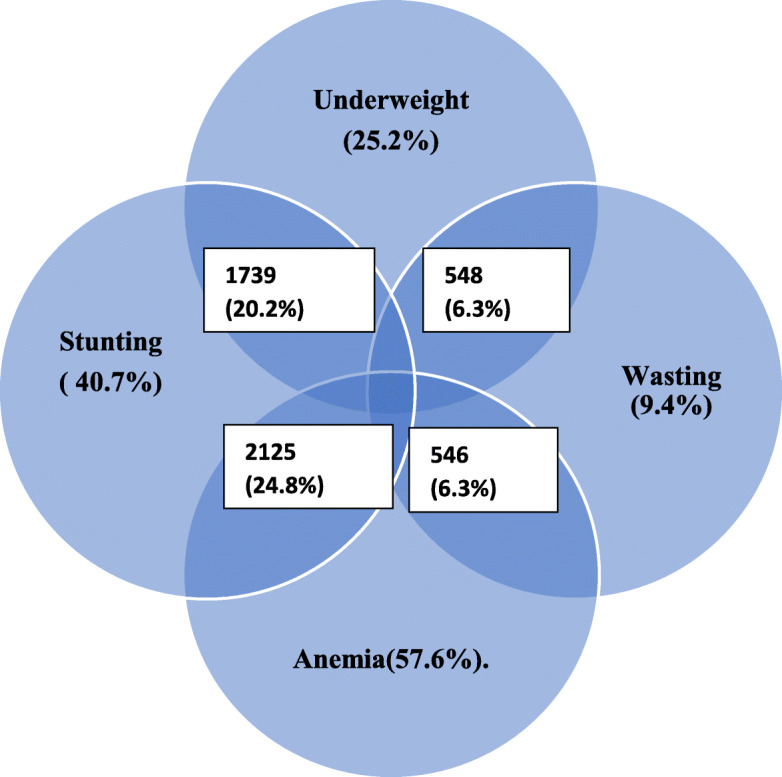
Venn diagram illustrating distribution of nutritional problems as well as concurrent nutrition deficits

In the secondary analysis, concurrent anemia and stunting (CAS) is the outcome of interest since the prevalence of children with concurrent stunting and anemia was the highest (24.8%.) compared to other pair-wise co-occurrences of the nutritional problems (Fig. 1), such as the concurrent stunting and underweight (20.2%.), Children with concurrent wasting and anemia, and wasting and underweight were each much lower (6%). Other possible co-occurrence (such as wasting and stunting; underweight and anemia) each had a smaller prevalence (not shown in the figure) The prevalence of CAS varied across population groups (Table 2).

**Table 2:** Results of Chi-square analysis for association between the explanatory variables and concurrent stunting and anemia (CAS), Ethiopia

Characteristics	No CAS	CAS	Chi-square (***p***-value)
**Maternal and child characteristics**			
**Sex of the child**			
Male	3251 (73.4)	1180 (26.6)	16.57 (0.000)
Female	3191 (77.2)	944 (22.8)	
**Age of the child**			
6-23 months	2262 (76.7)	687 (23.3)	20.93 (0.000)
24-35 months	1297 (71.1)	526 (28.9)	
Above 35 months	2884 (76.0)	911 (24.0)	
**Child’s birth order**			
First	1163 (77.1)	346 (22.9)	3.45 (0.033)
Second and above	5279 (74.8	1779 (25.2)	
**Age of mothers**			
15-24	1266 (71.8)	497 (28.2)	16.41 (0.000)
24-35	3504 (75.5)	1138 (24.5)	
35+	1673 (77.3)	490 (22.7)	
**Education of mothers**			
No education	4167 (72.8)	1553 (27.2)	69.63 (0.000)
Primary level	1788 (78.2)	499 (21.8)	
Secondary and above	488 (87.0)	73 (13.0)	
**Education of fathers**			
No education	3173 (72.9)	1182 (27.1)	54.46 (0.000)
Primary level	2491 (75.8)	797 (24.2)	
Secondary and above	779 (84.3)	145 (15.7)	
**Wealth index**			
Poorest	2792 (69.9)	1202 (30.1)	126.50 (0.000)
Poorer	1407 (76.9)	423 (23.1)	
Middle	2244 (81.8)	500 (18.2)	
**Type of family structure**			
Monogamous	5445 (75.6)	1757 (24.4)	1.43 (0.220)
Polygamous	672 (72.6)	253 (27.4)	
**Religion**			
Orthodox	2335 (79.1)	617 (20.9)	36.64 (0.000)
Others	4107 (73.2)	1507 (26.8)	
**Never breast fed the child**			
No	6204 (75.2)	2043 (24.8)	3.45 (0.150)
Yes	239 (74.7)	81 (25.3)	

^*^All analysis were weighted

### Chi-square analysis

In Table 3, the results of chi-square analysis show strong significant association between considered exposure variables and the outcome variable i.e., concurrent stunting and anemia. The table contains both background variables and childcare practice variables. Similarly, Table 4 presents bivariate mixed effect Poisson regression for multiple nutrition deficiency by background and some childcare practice variables.

**Table 3:** Unadjusted mixed-effects Poisson regression for the predictors of multiple nutritional deficiencies, Ethiopia

Characteristics^**a**^	RR (95 % CI)	***p***-values
**Sex of the child**		
Male		
Female	0.926 (0.891-0.963)	<0.001
**Child’s age**		
0-23 months		
24-35 months	1.001 (0.951-1.055)	0.960
36-59 months	0.769 (0.737-0.804)	<0.001
**Child’s birth order**		
First		
Second and above	0.703 (0.668-0.741)	<0.001
**Age of mother**		
15-24		
25-34	0.762 (0.724-0.802)	<0.001
35+	0.863 (0.812-0.916)	<0.001
**Education of mother**		
No education		
Primary level	1.037 (0.984-1.094)	0.174
Secondary and above	0.916 (0.833-1.007)	0.069
**Education of father**		
No education		
Primary level	0.923 (0.878-0.970)	0.002
Secondary and above	0.993 (0.925-1.066)	0.853
**Religion**		
Orthodox		
Others	0.864 (0.798-0.935)	<0.001
**Intimate partners violence/IPV**		
Yes		
No	1.005 (0.956-1.057)	0.835
**Wealth index**		
Poorer/poorest		
Middle	0.915 (0.856-0.978)	0.009
Richer/richest	0.824 (0.772-0.879)	<0.001
**Parity**		
0-3		
4-6	0.736 (0.702-0.771)	<0.001
6+	0.674 (0.638 -0.713)	<0.001
**Breast feeding**		
Never		
Breast feed	0.703 (0.635-0.779)	<0.001
**Intake of Iron supplement**		
Yes		
No	1.045 (0.967-1.131)	0.261
**Diet diversity score**	0.943 (0.924-0.962)	<0.001
**Child health service score**	1.127 (1.107-1.147)	<0.001
**Water, hygiene, and sanitation**	1.099 (1.075-1.125)	<0.001
**Mean maternal education at cluster level**	0.976 (0.950- 1.003)	0.082

^a^All analysis were weighted

**Table 4:** Multivariable mixed-effects Poisson regression for the predictors of multiple nutritional problem, Ethiopia

Characteristics	RR (95 % CI)	***p***-values
**Random-effects Parameters**		
Cluster/ EAs	0.249 (0.213-0.292)	<0.001
**Fixed effect**		
**Sex of the child**		
Male		
Female	0.906 (0.869-0.944)	0.007
**Child’s age**		
0-23 months		
24-35	2.031 (1.911-2.160)	<0.001
36-59	1.556 (1.461-1.657)	<0.001
**Child’s birth order**		
First		
Second and above	0.755 (0.706-0.807)	<0.001
**Age of mother**		
15-24		
25-34	1.009(.948-1.074)	0.777
35+	1.389 (1.274-1.513)	<0.001
**Education of mother**		
No education		
Primary level	0.896 (0.847-0.949)	<0.001
Secondary and above	0.721 (0.649-0.801)	<0.001
**Education of father**		
No education		
Primary level	0.938 (0.891-0.988)	0.016
Secondary and above	0.998 (0.924-1.079)	0.970
**Religion**		
Orthodox Christians		
Others	0.938 (0.866-1.015)	0.113
**Parity**		
0-3		
4-6	0.741 (0.698-0.786)	<0.001
6+	0.613 (0.566-0.664)	<0.001
**Breast feeding**		
Never		
Breast feed	0.894 (0.801-0.998)	0.046
**Diet diversity score**	0.972 (0.953-0.992)	0.007
**Child health service score**	1.099 (1.079-1.121)	<0.001
**Sanitation score**	1.080 (1.036-1.126)	<0.001
**Mean maternal education at cluster level**	0.978 (0.953-1.004)	0.103
Constant	0.128 (0.105-0.156)	<0.001
Variance for only random effect model:	.0327 (0.283-0.377)	<0.001
Number of groups/clusters: 589 Deviance/DF= 1.53		

Note: the covariate has a multiplicative effect of exp(b), which is denoted as RR, on the expected mean of number of nutritional deficiencies

### Multivariable mixed effect regression analysis for multiple nutritional problems

In Table 2, the Poisson regression for multiple nutritional deficits (stunting, underweight, wasting, and anemia) is presented. The table portrays the Rate Ratios (RR) along their respective confidence intervals and p- values. Fourteen variables appeared to be significantly associated with the outcome variables (the number of nutritional problems a child was reported to have). The model fit statistics showed that the model is not over dispersed and Poisson regression model is appropriate for the present analysis. The random effect was also significant (p < 0.001), showing considerable between group/ cluster variations. The covariate effect is interpreted as: for every one unit increase in the covariate, the covariate has a multiplicative effect of exp(b) (denoted as RR) on the expected mean of number of conditions.

Among the maternal and child factors, eight of them appeared as significant predictors of multiple nutritional problems. This includes sex of the child, age of the child, birth order, parity, maternal age, maternal education, paternal education, and wealth index. Holding everything else fixed, the expected mean of nutritional problems decreased among male children by 8% (RR = 0.925, 95% CI:0.877–0.975) compared to females. The expected mean of multiple nutritional challenges increases as the child’s age increases. The expected mean is higher for older children, and lower for children of higher birth order and mothers of higher parity. There was a significant decline in the expected mean of multiple nutritional problem for children with better parental education and living in affluent households.

Among the childcare/ behavioral factors, four variables were significant determinants of the outcome variable. The expected mean of multiple nutritional deficiency was lower for breastfed children compared to those never breastfed. For every one unit increase in diet diversity score of a child, the expected mean decreases by about 3% (RR = 0.972, 95% CI: 0.953–0.992). The expected mean increased for children from households with higher child health service utilization and hygiene and sanitation score.

In Table 5, results from mixed-effect logistic regression analysis on predictors of concurrent stunting and anemia are given. The significant predictors included four maternal and child health characteristics (sex of the child, age of the child, maternal and paternal education) ,two household-level predictor (religion and household wealth index), one behavioral factor (hygiene and sanitation score) and one community-level factor (mean maternal education at cluster level). In the adjusted analysis, holding everything fixed, the odds of CAS decreased by 17% (AOR = 0.826; 95%CI:0.736–0.927) for male children compared to female children. The likelihood of concurrent stunting and anemia was higher for older children compared to the younger ones (< 24 months). As expected, the likelihood of concurrent stunting and anemia decreased for mothers having at least secondary education and those living in more affluent households. The likelihood for a child to experience CAS increased by 1.10 times for every unit increase in improper hygiene and sanitation score.

**Table 5:** Multivariable mixed effect logistic regression for the risk factors of concurrent stunting and anemia, Ethiopia

Random effect	% and anemic n(%)*	AOR (95% CI)	***P***-value
Cluster/ Enumeration areas	NA	0.261 (0.182-0.373)	<0.001
**Fixed effect parameters**			
**Sex of the child**			
Male	944 (22.8)		
Female	1180 (26.6)	0.826 (0.736-0.927)	<0.001
**Child’s age (months)**			
0-23	129 (12.5)		
24-35	1085 (29.0)	6.548 (5.259-8.152)	<0.001
36-59	911 (24.0)	4.288 (3.432-5.358)	<0.001
**Age of mother**			
15-24	497 (28.2)		
24-35	1138 (24.5)	0.951 (0.818-1.105)	0.513
35+	911 (24.0)	0.854 (0.713-1.023)	0.086
**Education of mother**			
No education	1553 (27.2)		
Primary level	499 (21.8)	0.880 (0.757-1.023)	0.098
Secondary and above	73 (13.0)	0.640 (0.479-0.856)	0.003
**Education of father**			
No education	1182 (27.1)		
Primary level	797 (24.1)	0.884 (0.769-1.014)	0.079
Secondary and above	145 (15.7)	0.805 (0.649-0.999)	0.049
**Religion**			
Orthodox	617 (20.9)		
Others	1507 (26.8)	1.374 (1.172-1.610)	<0.001
**Wealth index**			
Poorer/poorest	1202 (30.1)		
Middle	423 (23.1)	0.732 (0..614-0.872)	<0.001
Richer/richest	500 (18.2)	0.636 (0.536-0.754)	<0.001
**Breast feeding**			
Yes	2043 (24.8)		
Never	81 (25.3)	0.935 (0..694-1.258)	0.656
**Diet Diversity Score/ DDS**	1 (0)**	0.971 (0.919-1.027)	0.304
**Sanitation score**	2 (1)**	1.118 (1.009-1.240)	0.033
**Health service utilization score**	1 (2)**	1.005(.956-1.057)	0.833
**Mean maternal education at cluster level**	4.3 (2.6)**	0.937 (0.898-0.979)	0.004
Constant	-	0.081 (0.052-0.126)	<0.001
Variance of the random-effect only model	-	0.376 (0.285-0.495)	<0.001

*All analysis were weighted ** Median and Interquartile range

## Discussion

The present study primarily aimed at examining the factors associated with the degree of overlap between the main nutritional problems of under-5 children: the standard anthropometric measures of wasting, stunting and underweight, as well as anemia. The study also aimed at assessing the key risk factors associated with co-occurrence of stunting and anemia among children aged 6–59 months in Ethiopia based on nationally representative data. It is noted that the proportion of children who were stunted, underweight or wasted was 38%, 25% and 9%, respectively. About 58% of the sample children were anemic. The prevalence of children concurrently stunted and anemic was 24.8%.

The findings indicate that two-thirds of children had at least one nutritional problem while those having two or more accounted for one-third. The prevalence of CAS was close to one-quarter of children. Though studies on multiple anthropometric deficits are few, a cross-country study reported a considerable proportion of multiple anthropometric deficits [6]. The reported high proportion of multiple nutritional deficiencies is of a great public health concern as it has huge impacts on the likelihood of child health and survival. McDonald and colleagues indicated considerable excess mortality in children who had concurrent wasting and underweight [6].

The expected mean of multiple nutritional problems was determined by a range of individual, household, and behavioral factors. The three proximate variables (hygiene and sanitation score, feeding practice and child health service utilization score) were found to exert strong influence on the expected mean of multiple nutritional deficiencies. Similarly, the mixed effect logistic regression witnessed significant association between eight background variables and CAS. Among the three key childcare practices, only hygiene and sanitation score had significant influence on CAS. The type of risk factors of CAS identified in this study have some resemblance with the recent findings of Shimeles and colleagues which used the same data set. Sex of the child, diet diversity, household wealth, and parental educational level were significant determinants in both studies. However, their study was based on only children of 6–23 months and primarily focused on assessing the dietary and nondietary associated factors [4]. The following discusses those explanatory variables which are common to both Mixed effect Poisson and Logit models.

Our findings showed that female children are less prone to multiple anthropometric deficiencies and CAS compared to males. Consistent with this finding, studies around the world found that boys were significantly more likely to experience concurrent wasting than girls [27]. A study on concurrent wasting and stunting based on DHS data of Senegal [28] found that boys were at higher risk of experiencing concurrent wasting and stunting compared to girls (RR = 1.61), which changes rapidly with age. Contrary to this finding, a recent study in Sri Lanka reported that female children had significantly higher rates of underweight and stunting compared to male [29]. The lack of a gender differential in adverse growth-stunting in Bangladesh was attributed to the absence of intra-household gender bias in feeding and health care for children [30]. The inconsistent findings could arise from variations in the sample size, method of analysis and seasonality of the data collected. However, the finding warrants the importance of considering a child’s gender during case finding and management/intervention of multiple anthropometric deficits.

It is also noted that the likelihood of multiple nutritional deficit and CAS increases as the age of the child increases. This finding agreed with studies conducted in Ethiopia and in Central African Republic [31, 32] and other developing countries [33].These studies claimed that stunting is less common in early infancy as most children are being breastfed [31–33]. The risk of impaired linear growth increases as breastfeeding is discontinued without adequate complementary feeding and with poor diet diversity [31, 34].

The effect of religion on early childcare and the nutritional status of children may be explained as noting that some religious practices and beliefs have an adverse influence on the consumption of some healthy foods, child feeding practices of mothers and dietary intake of children during early ages [29].

The expected mean of multiple nutritional problems and odds of CAS significantly decreased by a considerable amount as we compare children residing in households with poorest wealth quantiles to those in the richest quantiles. It is difficult, however, to compare these findings to other studies as wealth is measured differently. The few studies conducted in low-income countries across the different continents, based on asset-based wealth index, have inconsistent conclusions. For instance, a study in Thailand showed that underweight and stunting measured by Concentration Index (CI) were least equitably distributed among the lowest income groups [35]. Another study conducted in Brazil showed a decreasing trend of stunting inequality as well as an overall malnutrition rate with wealth [36]. Contrary to these findings, a study in Mexico reported that household poverty is not a necessary condition for children to experience anthropometric deficits [37]. A study in Ecuador found no evidence to indicate any relationship between economic inequality and the nutritional status of children [38].

Remarkable inequalities were found in the expected mean of multiple nutritional deficiencies and CAS based on parental education. Children born from parents with at least primary level of education had little chance of experiencing multiple nutritional deficiencies compared to those born from non-educated parents. The finding is consistent with earlier studies conducted in different parts of Ethiopia [39–42]. Recent studies in other parts of the world reached similar conclusion [43, 44]. It is well known that a minimum education provides women with a general awareness of how to utilize available resources for improvement of their own nutritional status and that of their families. Education may also help mothers be informed about nutritional values of food and understandings of child physical and mental growth [29]. Education may enable women to make independent decisions, and to have greater access to household resources that are important to nutritional status [45]. The presence of maternal autonomy is one of the most common and plausible explanations about the positive impacts of education on nutritional status. A gender analysis study in eastern parts of Indonesia where women are known to suffer from marginalization, reported the presence of very high levels of chronic child undernutrition (58% stunting and ~ 33% underweight) [46]. Maternal marginalization and depreciation generally pass through a wide range of pathways to affect child health outcomes. Poor autonomy may affect gender roles and intra-household food distribution, both of which may have a subtle impact on the nutrition of both women and young children [47].

While maternal education appears to exert a stronger influence on multiple nutritional deficiencies and CAS, the present study also showed a small but detectable role for paternal education. This is not surprising as some studies around the world have reported that educated fathers are more involved with issues of diet/ nutrition and parenting behaviors, which contribute to the overall health and well-being of their young children [48, 49]. Additionally, educated fathers provide a higher household income, more freedom and supports, higher social status and stability, and more opportunities for their wives and children [50, 51].

Positive associations were found between water and sanitation conditions and multiple nutritional deficiencies. This result was not a surprise since more than half of the households in the current survey reported open defecation and very poor access to water. Open defecation is more pervasive in rural areas. Similar results were obtained in several studies conducted in developing countries. For instance, a study in India found that the prevalence of undernutrition among children in low standard households increases twice as much as for children living in high standard households [52]. Unsanitary conditions are aggravated by poor access to improved water sources and latrines. Studies conducted in Sri Lanka, Sudan and Philippines reported that improved water and better-quality sanitation facilities resulted in significant improvement in health conditions of children [29, 53, 54]. A national survey in India underscores that caregiver’s reported practice of washing their hands with soap after defecation was associated with a 14% reduced risk of stunting among children aged 0–23 month [55]. The implication of this finding is that nutrition interventions should contain a framework broader than nutrition-specific interventions, by adequately addressing the combined water, household sanitation and personal hygiene.

The mixed-effect regression further showed two additional childcare variables (breastfeeding and diet diversity score) having significant association with CAS. Mothers who never breastfed had a child with a higher likelihood of experiencing CAS. There is ample accumulated evidence showing the important role of breastfeeding in the prevention of different forms of childhood malnutrition and micronutrient deficiencies [56, 57] More specifically, exclusive breastfeeding up to six-month of age has profound biological effects and important consequences on health and nutritional outcomes of children [56, 57]. The immunological properties of breast milk contribute to ensuring adequate nutritional status, proper growth and develop morbidity prevention capacity in child body [58].Late introduction of complementary feeding and unacceptably low diet diversity score in Ethiopia (> 90%) might have exacerbated the high prevalence of CAS .

In relation to breastfeeding, it is noteworthy to mention the strong significant association between diet diversity and multiple nutritional deficiencies. The expected mean of multiple nutritional deficiency decreases by about 3% for every one-unit increase in diet diversity. Since the present study indicated unacceptably low diet diversity (i.e., only 5% of the children consumed > 4 food groups) and the overall intake of animal protein was low, most of them may have become at higher risk of micronutrient deficiencies that can lead to chronic malnutrition and stunting. The reported poor diet in most Ethiopian households is mainly due to very high level of household hunger due to poor access or poor utilization or both [59, 60]. Consistent with this finding, a recent study in Ghana reported that dietary diversity causes improvements in child health among those under-5 [61]. Studies in other African countries such as Burkina Faso [62] reached a similar conclusion. Since dietary diversity was measured over a one-day recall period, the result should be interpreted cautiously. However, the finding implies the need for more thorough studies on the sociocultural and physical barriers to food consumption/diet diversity.

### Strength and limitations

The current study increases our understanding of the socioeconomic inequalities in multiple anthropometric deficiency and anemia among children in Ethiopia. The findings could prove useful on a national scale in assessing the progress in our fight against child undernutrition and serve as an important resource for the planning, targeting, monitoring, and evaluating of future health promotion programs. The study also has some methodological limitations worth mentioning. First, the DHS survey employed a cross-sectional design, where data on the exposure and outcomes were collected at a specific point in time. Second, because data were collected from mothers/caregivers, most of whom had no education, there are possibilities of omission, under-reporting, or improper reporting of important information. Under-reporting of these characteristics may generate inflated estimates and /or misclassification bias.

## Conclusion

This study provided evidence for unacceptably high prevalence of stunting, anemia and CAS with substantial socioeconomic disparities in Ethiopia. Given the fact that children with multiple anthropometric deficit and anemia are at a heightened risk of mortality, the risk factors identified in the present study will help prioritize child survival interventions. The study underscores the importance of improving parental education, household wealth, hygiene and sanitation conditions, promoting feeding practice and child health service utilization. Also, any nutrition sensitive and specific intervention should consider child’s characteristics such as his/her age, gender, and birth order. The finding further calls for aggressive actions in terms of prevention, coordinated case-finding, and treatment of children with multiple nutritional challenges.

## Data Availability

The datasets used for this study are made available from ICF international/DHS program at https://dhsprogram.com/data/Access-Instructions.cfm. Thus, administrative permissions were required to access the raw data from this organization .Public access to the database is open upon permission.

## Abbreviations

ANC: Antenatal Care
ARI: Acute Respiratory Infection
CAS: Concurrent Anemia and Stunting
CSA: Central Statistics Authority
DDS: Diet Diversity Score
DHS: Demographic and Health Surveys (DHS)
EDHS: Ethiopian Demographic and Health Surveys
GDP: Gross Domestic Product
IPV: Intimate Partners Violence
IYCF: Infant and Young Child Feeding
MOH: Ministry of Health
SDGs: Sustainable Development Goals
WHO: World Health Organization
